# Announcement: Eighth International Conference on Bioinorganic Chemistry

**DOI:** 10.1155/MBD.1996.53

**Published:** 1996


### ICBIC 8

YOKOHAMA '97

### EIGHTH INTERNATIONAL CONFERENCE ON BIOINORGANIC CHEMISTRY

to be held in the port city of Yokohama, Japan

***27 July - 1 August 1997***

**Introduction**

The Organizing Committee is pleased to invite all interested scientists to attend the Eighth International Conference on Bioinorganic Chemistry (ICBIC 8).The Conference will be held in the beautiful port city of Yokohama, Japan from 27 July to 1 August 1997. This Conference continues the biennial tradition, with conferences having been held in Florence, Italy (1983),Algarve, Portugal (1985), Noordwijkerhout, The Netherlands (1987), Boston, USA(1989), Oxford, UK (1991), San Diego, USA (1993), and Lübeck, Germany (1995).

An informal evening reception on Sunday, 27 July will precede the scientific sessions. Each morning will feature a plenary lecture followed by parallel sessions and poster presentations on a variety of contemporary research topics. The conference will end at noon, after the Friday morning sessions, 1 August.

The program will be organized around five daily plenary lectures, which will introduce topics to be expanded on by invited speakers in the subsequent sessions, and poster presentations.There will be ample opportunity for informal exchange of ideas.

**Tentative topics include:**

- Metal recognition, storage and transport
- Structure and functions of metalloenzyme active sites
- Industrial applications of bioinorganic chemistry
- Electron transfer
- Metals and nucleic acids
- Nitrogen and nitric oxide biochemistry
- Environmental bioinorganic chemistry
- Metals in medicine ... and more

**Invited Plenary Speaker**

- Jacqueline K. Barton *(California Institute of Technology, USA)*
- Robert Huber *(Max-Planck-Institut für Biochemie, Germany)*
- Kenneth D. Karlin *(The Johns Hopkins University, USA)*
- Teizo Kitagawa *(Institute for Molecular Science, Japan)*
- Peter J. Sadler *(University of London, UK)*

The Conference will continue the tradition of poster sessions to disseminate original and significant research in progress. There will be ample time for poster presentation because we plan to divide the poster session into two groups, both with two days for exhibition.
Conference organizers would like to encourage all participants to present posters. However so that there may be enough space for all, each participant will be limited to one poster
Formats required for poster presentations and for published abstracts will be with the second announcement. Abstracts will be published in the Journal of Inorganic Biochemistry for all pre-paid registrants.

**Social Events**
Get-together party on Sunday evening, 27 July.
Optional excursions on Wednesday afternoon, 30 July.
Official banquet on Thursday evening, 31 July.

The conference will take place at Pacifico Yokohama conference center, which is located on the waterfront near central Yokohama. Access to central Yokohama can be obtained via Narita express train or limousine bus from New Tokyo International Airport (Narita), taking 80 - 90 minutes. The conference site is also linked by subway with Shin-Yokohama station, where inkansen (bullet train) service is available, placing other important cities, including Osaka and Kyoto, within easy reach.
Yokohama, a tiny fishing village until the opening of its port in 1859, is now Japan's second largest city and is only 30 minutes by train from central Tokyo. Aiming towards a highly integrated international city in the next century, an urban redevelopment project is in progress, which has included the construction of Pacifico Yokohama.
Accommodation will be provided in hotels close to the conference site. The conference office will offer three categories. Arrangements may be made to arrive before and/or to stay after the meeting on a daily rate asis. Bed and breakfast-type accommodations in private homes will also be available.

**Organizing Committee**
*Chairman:* Masanobu Hidai (Tokyo U)
*Vice Chairmen:* Akira Nakamura (Osaka U), Hisanobu Ogoshi (Kyoto U), Osamu Yamauchi (Nagoya U)
Masahiko Fujino (Takeda Chem Ind Ltd), Masaaki Hirobe (Tokyo U), Tadashi Hirokane (Sumitomo ChemCo)
Yuzuru Ishimura (Keio U), Yoshinori Itokawa (Kyoto U), Nobuharu Izawa (Teijin Ltd)
Eiichi Kimura (Hiroshima U), Teizo Kitagawa (IMS), Makoto Komiyama (Tokyo U)
Kazuko Matsumoto (Waseda U), Yoshikazu Matsushima (Kyoritsu Coll Pharm), Isao Morishima (Kyoto U)
Yoshihiko Moro-oka (Tokyo Inst of Tech), Sun-ichi Murahashi (Osaka U), Yukito Murakami (Kyushu U)
Atsuo Nakanishi (CSJ), Hiroshi Ogino (Tohoku U), Masaji Ohno (Eisai Co)
Isashi Okawa (Kyushu U), Ishiro Okura (Tokyo Inst of Tech), Takeru Onoda (Mitsubishi Chem Co)
Tetsuo Osa (Tohoku U), Kazuhiko Saigo (Tokyo U), Isao Saito (Kyoto U)
Hiromu Sakurai (Kyoto Pharm U), Seiji Shinkai (Kyushu U), Yukio Sugiura (Kyoto U)
Kazuo Suzuki (Chiba U), Koji Tanaka (IMS), Eishun Tsuchida (Waseda U)

For receiving further announcements and/or information,
please write to:

**Professor Masanobu Hidai**
**Chairman, ICBIC 8**
**Department of Chemistry and Biotechnology**
**Graduate School of Engineering**
**The University of Tokyo**
**Hongo, Bunkyo-ku, Tokyo 113, Japan**

**Tel: +81-3-3812-211 l ext. 7261; Fax: +81-3-5800-6945**

